# Meeting Report: Metagenomics, Metadata and MetaAnalysis (M3) at ISMB 2010

**DOI:** 10.4056/sigs.1383476

**Published:** 2010-12-04

**Authors:** Dawn Field, Susanna Sansone, Edward F. DeLong, Peter Sterk, Iddo Friedberg, Renzo Kottmann, Lynette Hirschman, George Garrity, Guy Cochrane, John Wooley, Folker Meyer, Sarah Hunter, Owen White

**Affiliations:** 1 NERC Center for Ecology and Hydrology, Oxford, OX1 3SR, UK; 2 University of Oxford, Oxford e-Research Centre, Oxford, UK; 3 Department of Biological Engineering, Massachusetts Institute of Technology, Cambridge, MA 02138, USA; 4 Department of Microbiology, Miami University, Oxford OH 45056, USA; 5 Department of Computer Science and Software Engineering, Miami University, Oxford OH 45056, USA; 6 Microbial Genomics Group, Max Planck Institute for Marine Microbiology & Jacobs University Bremen, D-28359 Bremen, Germany; 7 Information Technology Center, The MITRE Corporation, 202 Burlington Road, Bedford, MA 01730, USA; 8 Department of Microbiology and Molecular Genetics, Michigan State University, East Lansing, Michigan 48824, USA; 9 European Molecular Biology Laboratory (EMBL) Outstation, European Bioinformatics Institute (EBI), Wellcome Trust Genome Campus, Hinxton, Cambridge CB10 1SD, UK; 10 University of California San Diego, 9500 Gilman Drive, La Jolla, CA 92093, USA; 11 Argonne National Laboratory, 9700 South Cass Avenue, Argonne, IL 60439, USA; 12 Institute for Genome Sciences, University of Maryland School of Medicine, Baltimore, MD 21201, USA

## Abstract

This report summarizes the proceedings of the first day of the *Metagenomics, Metadata and MetaAnalysis* (M3) workshop held at the Intelligent Systems for Molecular Biology 2010 conference. The second day, which was dedicated to the inaugural meeting of the BioSharing initiative is presented in a separate report. The Genomic Standards Consortium (GSC) hosted the first day of this Special Interest Group (SIG) at ISMB to continue exploring the bottlenecks and emerging solutions for obtaining biological insights through large-scale comparative analysis of metagenomic datasets. The M3 SIG included invited and selected talks and a panel discussion at the end of the day involving the plenary speakers. Further information about the GSC and its range of activities can be found at http://gensc.org. Information about the newly established BioSharing effort can be found at http://biosharing.org/.

## Introduction

Improved sampling of diverse environments (e.g. ocean, soil, sediment, and a range of hosts) combined with advances in the development and application of ultra-high throughput sequence methodologies are set to accelerate the pace at which new metagenomes are generated. The M3 & Biosharing SIG explored the latest concepts, algorithms, tools, informatic pipelines, databases and standards that are being developed to cope with the analysis of vast quantities of metagenomic data. It also sought to facilitate a broader dialogue among funders, journals, standards developers, technology developers and researchers on the critical issue of data sharing within the metagenomics community and beyond through the inaugural meeting of the BioSharing community (See http://biosharing.org for a list of participating communities). Through two days of invited and contributed talks, panel discussions, we aimed to highlight scientific advances in these fields and identify core computational challenges facing the wider community.

Because the pace of genomic and metagenomic sequencing projects [1] is rapidly increasing, and will only accelerate as the application of ultra-high-throughput methods becomes more wide spread, the role of standards is becoming ever more important to scientific progress and data sharing. The Genomic Standards Consortium (GSC) is an international working body with the mission of working towards richer descriptions of our collection of genomes and metagenomes through the development of standards and tools for supporting compliance and exchange of contextual information [2].

This report summarizes the proceedings of the *Metagenomics, Metadata and MetaAnalysis* (M3) Special Interest Group at ISMB 2010. Special Interest Group (SIG) meetings at the ISMB are a special way to bring together computational researchers interested in a particular topic. In establishing this new SIG, the GSC hopes to engage a wider range of bioinformatics researchers in thinking about standards. This SIG was named M3 to cover the important intersections between the ongoing explosion of data (**M**etagenomics) and the ever growing need to support richer stores of associated contextual data (**M**etadata) to improve our ability to interpret and comparing findings across large collections of independent studies (**M**etaAnalysis).

## M3 Session I: Metagenomics, Metadata and MetaAnalysis (M3)

The agenda of the M3 SIG was designed to focus on the intersections of science and standards and policy. The first day of the SIG was dedicated to metagenomic science. The first session contained five plenary talks by speakers championing each of an expanded set of five Ms or M5 (**M**etagenomics, **M**etadata, **M**etaAnalysis, **M**odels and **M**etaInfrastructure).

**Ed DeLong (Massachusetts Institute of Technology)** opened the session by talking about the promise of metagenomics for shedding light on community processes with his talk entitled *Understanding Microbes through Metagenomics.* This was followed by a talk from **Michael Ashburner (University of Cambridge)**, a founder of the Gene Ontology and co-coordinator of the OBO Foundry on the power of ontologies. **Phil Hugenholtz (Joint Genomes Institute)** gave an overview of the Microbial Earth project, an ambitious effort to sequence over 9,000 type strains to fill in the tree of life.

**Eric Alm (Massachusetts Institute of Technology)** and **Folker Meyer (Argonne National Laboratory)**, the final two plenary speakers focused on models and metainfrastructure. The vision of M5 is to bring democratized access to computation. The theme of how to bring the vision of an M5 platform to life was the theme of the final Panel session. Before lunch, **Dietlind Gerloff (UC Santa Cruz)** made a call for new collaborators in her flash talk *Sharing ‘raw’ experimental data between biological laboratories in a new agent-based infrastructure OpenKnowledge*.

## M3 Session II: Selected talks

After lunch, Session II continued with seven selected talks (from contributed abstracts) followed by a talk about the GSC to set the stage for the Panel session. The first pair of talks described software platforms. **Shulei Sun (UC San Diego)** talked about the latest release of the CAMERA 2.0 Workflow System. **Daniel H. Huson (Tübingen University)** then talked about functional analysis and comparison of meta-genomes, -transcriptomes and -proteomes using MEGAN4.

The next pair of talks described new approaches to understanding the content of metagenomic datasets. In the first talk, **Alexandre Lomsadze (Georgia Institute of Technology)** described an improved method for finding genes in metagenomic sequences. **Gail Rosen (Drexel University)** then talked about her new method of detecting Novel Species and Genera from Short Reads.

The focus then shifted to analysis of novel metagenomic datasets. The first talk described the generation of a dataset, the mobile gene pool metagenome from waste water, and was presented by **Antonia P. Mayer (University of Lausanne).** **Riccardo Percudani (University of Parma)** then described an analysis of human gut microbiome data that reveals an alternative pathway for urate degradation - a pathway for breaking down uric acid lost from the lineage leading to humans but potentially found in human gut bacteria.

The final pair of speakers brought the topic back around to the need for standards and efforts to build consensus. **Dirk Gevers (Broad Institute)** of the Human Microbiome Project then talked about the need to standardize protocols across laboratories. **Renzo Kottmann (Max Planck Institute Bremen)**, a member of the GSC Board, then closed out the session by describing the work of the GSC in a talk entitled *The Genomic Standards Consortium: A Community of Individuals*.

After the coffee break, **Folker Meyer** **(Argonne National Laboratory)** and **Sarah Hunter** **(European Bioinformatics Institute)** chaired an M5 panel discussion involving the plenary speakers from the first day. This discussion advanced a group vision of a collaborative, computational infrastructure to advance research in this area. More information on the GSC current strategy of the GSC about M5 can be found on the M5 homepage [3].

## Conclusions

In summary, the second M3 SIG meeting at ISMB brought together a wide range of researchers to share results and discuss the need for community-based solutions in the field of metagenomics. The GSC will continue to build on the M3 workshop series, with the next workshop held at July 2010 at biannual meeting of the International Society for Microbial Ecology. As a result of this workshop, the GSC helped to engage further with the international bioinformatics community, articulate more clearly the goals of the M5 roadmap and ultimately launch the BioSharing community [4]. The formation of the BioSharing community, including the GSC as a leading community, will undoubtedly aid the GSC in fulfilling its mission of improving data sharing and re-use in the specific domain of genomics and metagenomics.

## References

[1] Liolios K Chen IM Mavromatis K Tavernarakis N Hugenholtz P Markowitz VM Kyrpides NC The Genomes On Line Database (GOLD) in 2009: status of genomic and metagenomic projects and their associated metadata. Nucleic Acids Res 2010; 38(Database issue):D346-D354 10.1093/nar/gkp848 19914934 PMC2808860

[2] Field D Garrity GM Sansone SA Sterk P Gray T Kyrpides N Hirschman L Glockner FO Kottmann R Angiuoli S Meeting report: the fifth Genomic Standards Consortium (GSC) workshop. OMICS 2008; 12:109-113 10.1089/omi.2008.A3B3 18564915

[3] M5 home page: http://gensc.org/gc_wiki/index.php/M5

[4] Field D Sansone S DeLong EF Sterk P Friedman I Gaudet P Lewis S Kottmann R Hirschman L Garrity GM Meeting Report: BioSharing at ISMB. Stand Genomic Sci 2010; 3:300-303 10.4056/sigs/1403501 PMC3035313 21304729

